# Reproductive Health Outcomes Associated With Domestic Violence Among Iranian Women During the COVID‐19 Pandemic: A Cross‐Sectional Study

**DOI:** 10.1002/hsr2.72744

**Published:** 2026-06-30

**Authors:** Maryam Gharacheh, Fahimeh Ranjbar, Ebbie Kalan, Shahdokht Azadi, AbouAli Vedadhir, Tahereh Sadeghi

**Affiliations:** ^1^ Nursing and Midwifery Care Research Center, Health Management Research Institute Iran University of Medical Sciences Tehran Iran; ^2^ Department of Behavioral and Community Health, School of Public Health University of Maryland Maryland USA; ^3^ Department of Psychology, Gachsaran Branch Islamic Azad University Gachsaran Iran; ^4^ Department of Anthropology, Faculty of Social Sciences University of Tehran Tehran Iran; ^5^ Population Health Sciences University of Bristol Bristol UK; ^6^ Department of Pediatrics, School of Nursing and Midwifery, Akbar Hospital Mashhad University of Medical Sciences Mashhad Iran

**Keywords:** COVID‐19 pandemic, cross‐sectional studies, Iran, reproductive health, violence against women

## Abstract

**Background and Aims:**

Domestic violence (DV) is a major public health concern that negatively affects women's reproductive health. The COVID‐19 pandemic and related lockdowns may have exacerbated DV and its consequences. This study aimed to assess reproductive health outcomes associated with DV among married women in Iran during the COVID‐19 lockdown.

**Methods:**

A cross‐sectional study was conducted between November 2020 and September 2021, including 5317 married women attending healthcare centers across Iran. Data were collected on socio‐demographic characteristics, fertility‐related factors, and experiences of DV using the revised Conflict Tactics Scales (CTS2). Multivariate logistic regression models were applied to estimate adjusted odds ratios (AORs) and 95% confidence intervals (CIs) for the association between DV and reproductive health outcomes, adjusting for potential confounders.

**Results:**

Women who experienced DV were significantly more likely not to use contraceptives (AOR = 2.08; 95% CI, 1.61–2.70). DV was also associated with reduced likelihood of exclusive breastfeeding (AOR = 1.59; 95% CI, 1.21–2.10), lower participation in Pap smear screening (AOR = 1.43; 95% CI, 1.09–1.87), and higher risk of sexually transmitted infections (STIs) (AOR = 2.41; 95% CI, 1.33–4.38). These associations remained significant after adjusting for age, education, employment, and parity.

**Conclusion:**

DV during the COVID‐19 pandemic is strongly associated with adverse reproductive health outcomes among Iranian women, including unsafe contraceptive practices, inadequate breastfeeding, missed cervical cancer screening, and increased STI risk. These findings underscore the urgent need for public health interventions, including enhanced social and healthcare support, safety measures, and accessible reproductive health services for women experiencing DV, particularly during pandemics or periods of social restriction.

## Summary

- Domestic violence (DV) has detrimental effects on the reproductive health of Iranian women.
- The experience of DV is associated with the nonuse of contraceptives, inadequate exclusive breastfeeding practices, lack of Pap smear screenings, and an elevated risk of STIs.
- It is imperative to ensure sustained accessibility to social and healthcare services for women, emphasizing the enhancement of safety measures and the provision of support for individuals experiencing DV.

## 1 Introduction

The COVID‐19 pandemic has emerged as a rapidly evolving global public health crisis, unparalleled in speed and magnitude over the past century, resulting in significant mortality and morbidity [1]. This unprecedented global emergency has profoundly impacted various facets of life, encompassing the economy, policy, physical well‐being, and mental health. Developing nations have borne the brunt of this crisis, experiencing the greatest burden [2]. In times of crises, reproductive health assumes paramount importance, given the disruption of healthcare infrastructure and potential harm to families [3]. Comprising a broad spectrum of services, reproductive health care encompasses five fundamental components: provision of high‐quality family planning and infertility services; elimination of unsafe abortions; prevention and management of sexually transmitted infections (STIs), reproductive tract infections, cervical cancer, and other gynecological morbidities; enhancement of perinatal, antenatal, postpartum, and newborn care; and promotion of sexual health [4].

Domestic violence (DV) was operationally defined based on participants’ responses to the Persian version of the revised Conflict Tactics Scales (CTS2). DV was defined as the occurrence of at least one act of physical assault, psychological aggression, sexual coercion, or injury perpetrated by an intimate partner during the COVID‐19 lockdown period. Participants reporting one or more such acts (responses 1–6 on any relevant CTS2 subscale) were classified as “abused” (DV = 1), while those reporting no acts across all items were classified as “nonabused” (DV = 0). Violence against women is a significant manifestation of gender inequality that poses a substantial obstacle to advancing global reproductive health objectives [5]. The Eastern Mediterranean Region currently experiences the second‐highest global prevalence of violence against women, with rates reaching 37% [6]. In the context of the COVID‐19 pandemic, measures such as movement restrictions, isolation, and social distancing have disproportionately impacted the lives of women, exacerbating the adverse consequences [7]. Evidence from diverse sources and regions since the onset of the COVID‐19 outbreak has consistently indicated a surge in all forms of violence against women, particularly DV [8]. Notably, a recent study conducted in Iran revealed a 20% increase in DV prevalence during the COVID‐19 pandemic compared to prepandemic periods [9]. These distressing findings underscore the pressing need for comprehensive approaches aimed at addressing violence against women and fostering gender equality to effectively protect global reproductive health.

In domestic settings, a range of violations can occur, including intimate partner abuse. Consequently, during lockdowns, vulnerable women are compelled to coexist with their abusers. The imbalanced power dynamics within abusive households have the potential to escalate significantly during times of crisis. While the freedom of action for abusers increases, victims face restricted movement and limited capacity to cope or seek help due to ongoing exposure [10]. The implementation of quarantine policies leading to DV can have severe consequences, such as depression, anxiety, substance abuse, and even suicide [11, 12]. Research indicates that DV may contribute to adverse reproductive health behaviors and negative pregnancy outcomes through various mechanisms, thereby increasing the risks of unintended pregnancies, abortions, STIs [13, 14], and the exacerbation of chronic health issues stemming from stress related to trauma [15]. Consequently, DV presents a significant concern for women of reproductive age and poses a challenge for healthcare [16].

Meanwhile, the COVID‐19 pandemic has brought about significant changes that have had an impact on various aspects of reproductive health, such as couples’ sexual behavior, desire for pregnancy, and use of contraception [17, 18, 19, 20]. These changes have had wide‐ranging effects, including on more than 214 million women who wish to avoid pregnancy. These effects can be attributed to disruptions in supply chains, strained healthcare systems, and redirected resources caused by the pandemic [21, 22].

As of March 28, 2020, Iran had recorded 35,408 confirmed cases of COVID‐19 and a total of 2517 associated deaths. These figures positioned Iran at the 7th spot on the global list of confirmed cases. Consequently, a nationwide COVID‐19 lockdown was implemented in March and April 2020 to mitigate the spread of the virus [23]. Considering that DV is a public health consequence of the COVID‐19 crisis [10], it is imperative to adopt a multi‐sectoral approach to address this issue, with healthcare services playing a vital role in supporting affected women. A crucial component of the healthcare response is the identification of women who have experienced DV and facilitating their access to appropriate support services to enhance their safety and promote their reproductive health outcomes [24]. Nonetheless, only a small number of studies have explicitly linked DV to maternal outcomes during the COVID‐19 pandemic [25, 26, 27], and empirical data on the impact of DV on reproductive health outcomes among Iranian women in the pandemic period remain scarce. Furthermore, understanding of how pandemic‐related disruptions to health services affect access to prenatal care, and safe abortion within the DV context is limited. There is also a paucity of evidence to guide clinicians and policymakers on effective screening, referral, and integrated care pathways for DV within reproductive health services during public health emergencies. Addressing these gaps will enable healthcare providers to identify at‐risk women, ensure timely access to psychosocial and medical support, and adapt reproductive health services to pandemic constraints. Therefore, the findings could inform screening protocols, referral networks, and resource allocation in Iran and in similar settings facing public health emergencies. The objective of this study was to evaluate the reproductive health outcomes associated with DV among Iranian women during the COVID‐19 pandemic.

## 2 Methods

### 2.1 Study Design and Participants

This is part of an interview‐based cross‐sectional study was conducted between November 2020 and September 2021, involving 5317 married women who attended healthcare centers in five cities in Iran: Tehran, Tabriz, Mashhad, Shiraz, and Ahvaz (with a response rate of 92.58%). Data collection was conducted by trained interviewers across the five selected cities. A total of interviewers, with backgrounds in [midwifery, public health, nursing], were involved in the study. All interviewers received standardized training before data collection, which included instructions on administering the questionnaire, ethical considerations, and ensuring confidentiality. To maintain consistency across study sites, a uniform protocol was followed, and regular supervision was performed throughout the data collection process.

### 2.2 Sample Size Calculation

The sample size was determined using the following formula:

$$\text{Sample}\;\text{size}\;=\frac{\frac{z^{2}\times p(1-p)}{e^{2}}}{1+\left(\frac{z^{2}\times p(1-p)}{e^{2}N}\right)},$$

where *N* represents the population size, *e* = 0.015 (1.5%), and *z* = 1.96. Using a conservative estimate *p* = 0.5 to maximize the required sample size, and with a population size N = 20,000,000, the initial calculation yields *n*0 ≈ 4,268. Finite population correction (FPC) was considered but deemed negligible given the large sampling frame (*N* ≫ n0). Therefore, *n* ≈ n0 ≈ 4268. To account for anticipated nonresponse and incomplete questionnaires, we inflated the sample by 20%: unadjusted = *n*/(1 − 0.20) ≈ 4268/0.80 ≈ 5335.

Thus, the target sample size was 5335 respondents. Actual recruitment and final sample were as follows: approached 5892 women; agreed to participate 5455; completed questionnaires 5317; final analytic sample 5317.

### 2.3 Sampling Frame and Design

The sampling frame comprised women presenting for routine reproductive health and related services at participating healthcare centers. A multistage sampling design was employed to select study sites and participants. At the first stage, the country was divided into five regions according to the Ministry of Interior's regional classification, and one province was randomly selected from each region. In the second stage, within each selected province the capital city was subdivided into three districts‐northern, southern, and central. A list of healthcare centers within each district was compiled, and two centers per district were randomly selected. In the third stage, within each selected center, participants were consecutively sampled for inclusion in the study. Within‐center recruitment involved inviting eligible women to participate until the target sample size was reached. This design facilitated regional representation through a cluster‐based approach and sequential enrollment within centers to optimize feasibility while preserving sample integrity.

Eligible participants included ‘Iranian women who had been married for at least 1 year before the study and were living with their husbands in the same household. Exclusion criteria included women who were unable or unwilling to provide informed consent, those with severe physical or mental conditions preventing participation, and women who were temporarily absent from the household during the study period.

The research commenced following approval from the University Ethics Committee under the code number REDACTED and upon presenting an official letter of introduction from the relevant authorities in “REDACTED” University of Medical Sciences. Before the commencement of the interviews, the eligible women provided signed written informed consent. During the interviews, which took place in private rooms at the healthcare centers, the presence of the husband was not permitted to ensure confidentiality. To protect the participants’ anonymity, no names were recorded on the questionnaire. Participation in the study was voluntary, and participants retained the right to withdraw at any point during the research process.

### 2.4 Measures

*Socio‐demographic characteristics* were assessed, including the age of woman and her spouse, the age of the woman and her spouse at the time of marriage, length of marriage, education level, employment status, monthly family income, number of previous marriages for the woman and her spouse, as well as the spouse's smoking, addiction, and mental illness. Fertility‐related characteristics were also examined, such as the number of pregnancies (gravidity and parity), history of abortions, the number of children, and various reproductive health outcomes, including pregnancy intentions, mode of delivery, breastfeeding patterns, contraceptive use, cervical cancer screening, and history of STIs. In this study, patient health records were utilized to assess the reproductive health outcomes. The measurement of pregnancy intentions was based on the dichotomy of intended or unintended pregnancy. An unintended pregnancy was operationally defined as either unwanted, signifying a situation where the pregnancy occurred when no children or no more children were desired, or mistimed, indicating a situation where the pregnancy occurred earlier than desired. Regarding the mode of delivery, the study considered two categories: normal vaginal delivery (NVD) and cesarean section (CS). The patterns of breastfeeding were categorized as exclusive breastfeeding, mixed feeding (combining breastfeeding with formula feeding), or exclusive formula feeding. Contraception use was classified into two groups: nonuse and use of various methods, including intrauterine devices, oral contraceptive pills (OCP), injectable hormones, condoms, tubal ligation, and vasectomy. Cervical cancer screening was assessed by determining whether individuals had undergone Pap smear tests or not. Additionally, the study aimed to identify the participants’ history of STIs, which encompassed Herpes simplex, human papillomavirus (HPV), human immunodeficiency virus (HIV), Hepatitis B, Hepatitis C, as well as other common STIs such as trichomoniasis and gonorrhea.

*DV* was measured using the Persian version of revised CTS2 [28], which is a 36‐item questionnaire consisting of subscales that assess different forms of DV, including physical assault (12 items), psychological aggression (8 items), sexual coercion (4 items), injury (6 items), and negotiation (6 items) [29]. For each item, participants reported the frequency of specific behaviors during the lockdown period, selecting from response options ranging from “once” to “> 20 times,” with additional options for incidents that occurred before the referent period or never [28]. Following established methodology, a dichotomous variable was created to classify exposure. A participant was assigned a score of one (DV = 1, “abused”) if they reported the occurrence of at least one act of violence (responses 1–6) on any relevant subscale of the CTS2. Conversely, a score of zero (DV = 0, “nonabused”) indicated no reported acts of violence across all items [30]. The reliability of the instrument was established using Cronbach's alpha coefficient (*α* = 0.89). This scale has been previously validated among the “REDACTED”‐ian population [29].

### 2.5 Data Analysis

Continuous variables were reported as means with standard deviations (SD), while categorical variables were expressed as frequencies and percentages. Independent sample *t*‐tests and Chi‐square tests were employed to compare continuous and categorical variables between the two groups, respectively. Subsequently, multivariate regression models were utilized to identify factors associated with DV. Adjusted odds ratios (AORs) and 95% confidence intervals (CIs) were reported. Data analysis was conducted using SPSS software, version 22.0 (Armonk, NY: IBM Corp), and a p‐value of less than 0.05 was considered statistically significant.

It should be noted that the STROBE checklist was utilized as a guide in this study.

## 3 Results

An analysis of the study sample, consisting of 5317 women of reproductive age (15–49 years), was conducted. The mean ages of the women and their husbands were 34.37 ± 9.66 years and 38.84 ± 11.00 years, respectively. Of the total sample, 439 women (8.3%) were pregnant at the time of the interview. During the COVID‐19 lockdown, 74.7% of the women reported experiencing at least one type of DV [31]. Statistically significant differences were observed between abused and nonabused women across several demographic variables. Specifically, women who experienced DV were significantly younger than their nonabused counterparts (33.79 vs. 36.23 years). Furthermore, the mean duration of marriage was significantly shorter among abused women (11.72 years) compared to nonabused women (14.35 years). Additional socio‐demographic characteristics can be found in Table 1. Out of the total sample, 1168 (22.0%) women were affected by COVID‐19 infection, with a higher infection rate observed among the abused women compared to the nonabused group (22.9% vs. 18.9%; *p* = 0.002) (Tables 1 and 2).

**Table 1:** Demographic characteristics of the participants (*n* = 5317).

Variable	Nonabused women (*n* = 1343)	Abused women (*n* = 3974)	Mean difference (95% CI)	*p* value	Cohen's *d* (approx.)
Women's age (year)	36.29 ± 10.23 36 (29–43)	33.72 ± 9.38 33 (26–39)	2.57 (1.98–3.17)	< 0.001	0.25
Man's age (year)	40.91 ± 10.98 38 (32–45)	38.14 ± 10.93 36 (30–42)	2.77 (2.09–3.45)	< 0.001	0.26
Marriage duration (year)	14.42 ± 11.30 12 (6–22)	11.61 ± 9.63 10 (4–18)	2.81 (2.18–3.43)	< 0.001	0.27
Gravidity	2.19 ± 1.69 2 (1–3)	1.79 ± 1.49 2 (1–3)	0.40 (0.30–0.50)	< 0.001	0.25
Abortion	0.32 ± 0.70 0 (0–0)	0.33 ± 0.69 0 (0–0)	–0.016 (–0.06–0.03)	0.461	0.02
Living children	1.83 ± 1.41 2 (1–2)	1.40 ± 1.20 1 (1–2)	0.43 (0.35–0.51)	< 0.001	0.33

*Note: p*‐values were calculated using the independent samples *t*‐test for continuous variables. Continuous variables are presented as mean ± SD and median (Q1–Q3) to describe both central tendency and distribution.

**Table 2:** Demographic and socioeconomic characteristics of nonabused and abused women.

Variable	Nonabused women (%)	Abused women (%)	*χ* ^2^/*p* value*
Women's education			*χ* ^2 ^= 3.003, *p* = 0.223
Illiterate	12 (0.9)	69 (1.7)	
Secondary school	664 (49.4)	2253 (56.7)	
Academic	534 (39.8)	1785 (44.9)	
Total	1343 (100)	3974 (100)	
Husbands’ education			*χ* ^2 ^= 5.066, *p* = 0.079
Illiterate	11 (0.8)	74 (1.9)	
Secondary school	627 (46.7)	2147 (54.0)	
Academic	572 (42.6)	1886 (47.4)	
Total	1343 (100)	3974 (100)	
Women's occupation			*χ* ^2 ^= 10.79, *p* = 0.005
Unemployed	799 (59.5)	2346 (59.0)	
Employed	401 (29.9)	1194 (30.1)	
Retired	10 (0.7)	67 (1.7)	
Total	1343 (100)	3974 (100)	
Husband's occupation			*χ* ^2 ^= 15.15, *p* = 0.001
Unemployed	61 (4.5)	346 (8.7)	
Employed	1081 (80.5)	3542 (89.1)	
Retired	68 (5.1)	219 (5.5)	
Total	1343 (100)	3974 (100)	
Economic status			*χ* ^2 ^= 138.42, *p* = 0.001
Low	162 (12.1)	1244 (31.3)	
Middle	885 (65.9)	2455 (61.8)	
High	163 (12.1)	408 (10.2)	
Total	1343 (100)	3974 (100)	

* The *p*‐value was calculated by the *χ* ^2^ test.

Our results revealed a significant difference in the rates of contraceptive use, exclusive breastfeeding, Pap smear screening, and contracting STIs between abused and nonabused women, which were more pronounced in former group (Table 3). While the rate of unintended pregnancies was higher among abused women compared to nonabused women (20.1% vs. 16.4%), the difference did not reach statistical significance (*p* = 0.098). The utilization of contraceptive methods varied among women exposed to DV, with lower rates of hormonal contraceptive and condom use, and higher rates of withdrawal method and contraceptive nonuse observed among abused women. Approximately 5% of women in our study had an STI, including HPV (1.1%), HSV (0.9%), HIV (0.5%), HBV (0.3%), HCV (0.3), and other infections such as Trichomoniasis (1.7%).

**Table 3:** Distribution of pregnancy intention, contraceptive use, breastfeeding, Pap smear screening, and STIs among nonabused and abused women.

	Nonabused women (*n* = 1210) (%)	Abused women (*n* = 4107) (%)	*χ* ^2^/*p* value
Pregnancy intention			χ^2 ^= 1.890, *p* = 0.098
unintended pregnancies	55 (16.4)	123 (20.1)	
Intended pregnancies	280 (83.6)	490 (79.9)	
Total	335 (100)	613 (100)	
Contraceptive use			*χ* ^2 ^= 32.86, *p* = 0.001
None‐use	412 (40.4)	1824 (50.6)	
Use	607 (59.6)	1781 (49.4)	
Total	1019 (100)	3605 (100)	
Exclusive breastfeeding			*χ* ^2^ = 13.17,*p* = 0.001
No	147 (28.0)	305 (37.6)	
Yes	378 (72.0)	506 (62.4)	
Total	525 (100)	811 (100)	
Pap smear screening			χ^2^=24.44, *p* = 0.001
No	504 (41.7)	2044 (49.8)	
Yes	705 (58.3)	2063 (50.2)	
Total	1209 (100)	4107 (100)	
Having STIs			*χ* ^2 ^= 11.95, *p* = 0.001
No	1174 (97.0)	3885 (94.6)	
Yes	36 (3.0)	222 (5.4)	
Total	1210 (100)	4107 (100)	

Furthermore, our regression analysis demonstrated a significant association between DV during the COVID‐19 lockdown and various adverse reproductive health outcomes. Specifically, DV was significantly associated with contraception nonuse (AOR = 2.08; 95% CI, 1.61–2.70), lack of exclusive breastfeeding (AOR = 1.59; 95% CI, 1.21–2.10), lack of Pap smear screening (AOR = 1.43; 95% CI, 1.09–1.87), and increased likelihood of having STIs (AOR = 2.41; 95% CI, 1.33–4.38) (Table 4).

**Table 4:** Logistic regression analysis of reproductive health outcomes associated with domestic violence.

Variable	Reference (*n*)	Category (*n*)	AOR	95% CI for OR	*p* value
Women's age (year)	—	—	1.01	0.98–1.06	0.474
Husbands’ age (year)	—	—	0.97	0.94–1.01	0.129
Duration of marriage (year)	—	—	0.997	0.967–1.028	0.848
Gravidity	—	—	1.24	1.01–1.51	0.040
Living children	—	—	0.80	0.62–1.05	0.104
Women's occupation	Unemployed (nonabused: 799, abused: 2346)	—	—	—	—
Employed (nonabused: 401, abused: 1194)	—	0.82	0.60–1.12	0.213	No
Retired (nonabused: 10, abused: 67)	—	0.76	0.12–4.68	0.765	No
Husbands’ occupation	Unemployed (nonabused: 61, abused: 346)	—	—	—	—
Employed (nonabused: 1081, abused: 3542)	—	0.43	0.22–0.84	0.014	Yes
Retired (nonabused: 68, abused: 219)	—	0.47	0.18–1.21	0.119	No
Economic status	Low (nonabused: 162, abused: 1244)	—	—	—	—
Middle (nonabused: 885, abused: 2455)	—	0.31	0.23–0.42	0.001	Yes
High (nonabused: 163, abused: 408)	—	0.13	0.09–0.21	0.001	Yes
Contraception use	Nonuse (nonabused: 412, abused: 1824)	Use (nonabused: 607, abused: 1781)	2.09	1.61–2.71	0.001
Exclusive breastfeeding	Nonexclusive (nonabused: 147, abused: 305)	Exclusive (nonabused: 378, abused: 506)	1.60	1.21–2.11	0.001
Pap smear screening	No (nonabused: 504, abused: 2044)	Yes (nonabused: 705, abused: 2063)	1.44	1.10–1.88	0.008
Having STIs	No (nonabused: 1174, abused: 3885)	Yes (nonabused: 36, abused: 222)	2.42	1.33–4.38	0.004

*Note:* The regression analysis was adjusted for demographics characteristics. Odds ratios are significant at alpha = 0.05.

Abbreviations: AOR, adjusted odds ratio; CI, confidence interval.

## 4 Discussion

This is a large‐scale study conducted in “REDACTED,” a developing country that contributes to the growing body of literature on reproductive health outcomes associated with DV during the COVID‐19 lockdown. Our study demonstrates that DV during the COVID‐19 lockdown was associated with adverse reproductive health outcomes. Specifically, women who experienced DV were less likely to use contraceptive methods and showed altered patterns of contraceptive use, including lower use of reliable methods and reduced condom usage. These findings align with the notion that DV undermines women's autonomy over fertility and creates barriers to accessing sexual reproductive health (SRH) services, a phenomenon observed in various settings, even in developed countries [32], reflecting the shifts in power dynamics and service accessibility during crises. Silverman and Raj [33] have also reported that perpetrators’ control over reproductive decisions can restrict access to family planning and SRH services, a pattern that appears intensified during the pandemic. A systematic review has highlighted that the disruptions caused by the COVID‐19 pandemic have had a detrimental impact on access to SRH services. This includes limited access to contraceptives, reduced availability of HIV/STI testing, changes in sexual behaviors, and alterations in pregnancy intentions [34]. These findings reflect constraints imposed by public health policies and quarantine restrictions that can impede access to SRH services on a population level. More specifically, our findings suggest that contraceptive use among abused women is reduced, which could be due to physical limitations in accessing services, fear of contacting providers during quarantine periods, or diminished decision‐making power under the pressures related to DV. However, our study did not identify significant statistical differences in unintended pregnancies, which may indicate broader systemic barriers to contraceptive access that warrant further investigation for applicability to macro‐level policies. Furthermore, a more detailed exploration of these findings necessitates a distinction between service availability/accessibility and actual service utilization to elucidate a more precise causal relationship with DV.

Consistent with prior research, abused women in our sample were less likely to engage in exclusive breastfeeding, and a lack of exclusive breastfeeding was associated with a higher risk of DV. A systematic review has linked violence exposure to shortened breastfeeding duration and earlier termination [35]. Additionally, another study reported correlations between DV and suboptimal breastfeeding practices in the first 6 months of life [36]. The impact of DV on breastfeeding is multifactorial, encompassing direct physical and psychological effects, diminished social support, and negative body image or mood disorders [35]. Abused women may also resort to maladaptive coping mechanisms, which can compromise their ability to breastfeed may be compromised due to physical, psychological, or cognitive impairments [37]. Prenatal and postnatal support, including counseling and education, has been shown to improve breastfeeding outcomes [38]. However, lockdown‐related restrictions hindered access to these essential services. Healthcare providers have also faced difficulties in reaching out to women due to restricted movement, social distancing measures, and limited communication channels [8]. These concerns are particularly important in crisis contexts such as pandemics. The lack of social and health supports can have serious consequences for the mental and physical health of women experiencing DV, underscoring the need for policies and programs designed to provide such supports. In addition, creating appropriate platforms for women to access health and counseling services can play an important role in improving breastfeeding outcomes and reducing the effects of DV. DV was also associated with lower Pap smear uptake. Prior studies have reported similar patterns, with violence exposure linked to reduced cervical cancer screening. For instance, Gandhi et al. [39] reported that middle‐aged women who had experienced physical and/or sexual violence had an 87% reduced likelihood of being up to date on Pap smears. Additionally, Cadman et al. [40] found that sexual abuse acts as a barrier to attending cervical screening appointments. The COVID‐19 pandemic further exacerbated barriers to screening, with emotional and psychological factors playing significant roles in limiting preventive behaviors [41]. In some cases, women may avoid pelvic examinations due to traumatic associations [39]. Therefore, there is an increasing need for support and educational programs to raise awareness and help women face these challenges so that they can access the health services they need and protect their health during crises.

We observed a significant association between DV and higher STI prevalence, with women who had STIs being more likely to report experiencing DV. A possible explanation is that lower condom uses in abusive relationships, together with gender power imbalances and risk‐taking behaviors by partners, elevates STI risk. Cultural contexts may shape these dynamics; for example, in some settings, women may be pressured into unprotected sex to prevent conflict [42]. Cross‐cultural evidence supports the link between DV and higher STI symptoms among women experiencing DV, underscoring the need for integrated DV and sexual health interventions. The observed association emphasizes that vulnerability to STIs among DV‐affected women is likely mediated by multiple interacting factors, including condom use, partner behaviors, and broader gender norms. A study conducted in Nepal reported that approximately one‐fourth of women who experienced physical and sexual violence had contracted STIs within the past 12 months [43]. Additionally, Bondade et al. [44] discovered that women who experienced DV exhibited a higher prevalence of STI symptoms compared to those who did not. The gender power imbalance between men and women, coupled with the risky behaviors of abusive partners, such as engaging in multiple sexual partnerships, further exacerbates women's vulnerability to STIs [45]. Therefore, it is recommended that STI screening be conducted for women experiencing DV, and that those diagnosed with STIs be evaluated for potential abusive relationships. We also found a higher rate of COVID‐19 infection among the abused women. These findings highlight the importance of recognizing the intersection of gender inequality with the transmission dynamics of infectious diseases such as COVID‐19. DV can significantly impact community spread. Specifically, incidents of sexual abuse and unprotected sexual intercourse can escalate the risk of exposure to infectious diseases. Furthermore, some abusers exploit the fear surrounding the COVID‐19 pandemic to exert power through psychological violence, employing tactics such as threats of infection, restricting access to hygiene supplies, and impeding testing and vaccination efforts [46]. Collectively, these actions contribute to increased vulnerability among women, not only in terms of acquiring COVID‐19 but also in terms of potential post‐COVID‐19 complications, including long‐term physical and mental health conditions.

While the current research represents a large‐scale study, it is important to acknowledge certain limitations. First, data collection was confined to urban health centers located in major cities, which restricts the generalizability of the findings to rural areas. Additionally, due to the cross‐sectional design of the study, it is not possible to establish causal relationships between DV and reproductive health outcomes. However, this study sets the groundwork for future longitudinal studies with larger sample sizes.

Moreover, our reliance on patient health records to assess the reproductive health outcomes restricted the scope of our results. The association between DV during the lockdown and STIs should be interpreted with caution as some STIs may have predated the lockdown period. Furthermore, it is crucial to consider the potential underreporting of DV due to societal stigma, feelings of shame, and other socio‐cultural factors. Nonetheless, in an effort to mitigate response or reporting bias, female healthcare professionals from the local community were enlisted to conduct interviews with participants in private settings. This approach aimed to create a more conducive environment for open and honest communication about sensitive experiences.

## 5 Conclusions

Our study findings indicate that there is a significant association between DV and various reproductive health factors such as contraception nonuse, lack of exclusive breastfeeding, absence of Pap smear screening, and increased risk of contracting STIs. Therefore, it is crucial for authorities and support networks to implement appropriate strategies in response to violence against women during pandemics. Since healthcare systems often serve as the primary point of contact for abused women, and healthcare professionals are trusted sources of help and information [47], it is essential to provide training programs for healthcare professionals to effectively identify and address cases of DV during pandemics.

Based on our findings, DV during the lockdown had a negative impact on breastfeeding exclusivity. Consequently, it is necessary to develop strategies that can support mothers in such situations following childbirth. Remote service delivery methods, such as leveraging mobile health (mHealth) platforms, offer an opportunity to engage with women and provide them with education and counseling support. Considering the intricate nature of this association, further research is warranted to explore the reproductive health outcomes associated with DV. Future studies should also include vulnerable groups, particularly pregnant women experiencing DV, to fully understand the unique challenges they face and develop targeted interventions to ensure their well‐being.

## Author Contributions

**Maryam Gharacheh:** conceptualization, funding acquisition, formal analysis, investigation, project administration, supervision writing – original draft. **Fahimeh Ranjbar:** conceptualization. **Ebbie Kalan:** software, methodology, validation. **Shahdokht Azadi:** data collection. **AbouAli Vedadhir:** conceptualization, methodology. **Tahereh Sadeghi:** conceptualization, writing – original draft, writing – review and editing.

## Ethics Statement

In the present study, all procedures were conducted adhering to the pertinent guidelines and regulations, particularly the Declaration of Helsinki. Ethical approval was obtained from the Ethics Committee of Iran University of Medical Sciences (IR.IUMS.REC.1399.362).

## Consent

Before the study, informed consent was procured from all participants. Written informed consent was obtained from a parent and/or a legal guardian if participants were under the age of 16 years. The illiterates’ informed consent method was approved by the Ethics Committee of Iran University of Medical Sciences. All participants were explicitly informed about their voluntary participation and their right to withdraw from the study at any stage. To ensure confidentiality and privacy, questionnaires were anonymously completed, employing only unique identifiers for each respondent.

## Conflicts of Interest

The authors declare no conflicts of interest.

## Transparency Statement

Tahereh Sadeghi affirms that this manuscript is an honest, accurate, and transparent account of the study being reported; that no important aspects of the study have been omitted; and that any discrepancies from the study as planned (and, if relevant, registered) have been explained.

## Data Availability

The data that support the findings of this study are available from the research deputy of Iran University of Medical Sciences, but restrictions apply to the availability of these data, which were used under license for the current study, and so are not publicly available. Data are however available from the corresponding author upon reasonable request and with the permission of the research deputy of Iran University of Medical Sciences.

## References

[1] S. Meherali , B. Adewale , S. Ali , et al., “Impact of the COVID‐19 Pandemic on Adolescents’ Sexual and Reproductive Health in Low‐ and Middle‐Income Countries,” International Journal of Environmental Research and Public Health 18, no. 24 (2021): 13221. 34948829 10.3390/ijerph182413221 PMC8701118

[2] S. Shahyad and M. T. Mohammadi , “Psychological Impacts of Covid‐19 Outbreak on Mental Health Status of Society Individuals: A Narrative Review,” Journal of Military Medicine 22, no. 2 (2020): 184–192.

[3] M. Shariati , R. Babazadeh , S. A. Mousavi , and K. M. Najmabadi , “Iranian Adolescent Girls’ Barriers in Accessing Sexual and Reproductive Health Information and Services: A Qualitative Study,” Journal of Family Planning and Reproductive Health Care 40, no. 4 (2014): 270–275. 25183530 10.1136/jfprhc-2013-100856

[4] World Health Organization , Reproductive Health Strategy to Accelerate Progress Towards the Attainment of International Development Goals and Targets (World Health Organization, 2004). 10.1016/s0968-8080(05)25166-2 16035592

[5] A. S. N. Nia , M. Dolatian , B. H. P. Azghadi , A. Ebadi , and A. A. Baghban , “Domestic Violence and Its Association With Domains of Reproductive Health in Women: A Systematic Review,” Journal of Mazandaran University of Medical Sciences 27, no. 158 (2018): 205–217.

[6] WHO . *Preventing and Responding to Gender‐Based Violence Against Women and Girls in the Eastern Mediterranean Region*, Accessed June 1, 2021, https://www.emro.who.int/violence-injuries-disabilities/violence-news/prevention-and-response-to-gender-based-violence.html.

[7] UN Women , Policy Brief: The impact of COVID‐19 on women 2020, Accessed June 1, 2021, https://www.unwomen.org/sites/default/files/headquarters/attachments/sections/library/publications/2020/policy-brief-the-impact-of-COVID-19-0n-women.

[8] UN Women , Impact of COVID‐19 on Violence Against Women and Girls and Service Provision: UN Women Rapid Assessment and Findings 2020, Accessed June 1, 2021, https://www.unwomen.org/en/digital-library/publications/2020/05/impact-of-covid-19-on-violence-against-women-and-girls-and-service-provision.

[9] R. Fereidooni , J. Mootz , R. Sabaei , et al., “The COVID‐19 Pandemic, Socioeconomic Effects, and Intimate Partner Violence Against Women: A Population‐Based Cohort Study in 2020, Iran,” American Journal of Public Health 113, no. 2 (2021): 228–237. 10.2105/AJPH.2022.306839 PMC9850608 36302221

[10] L. E. B. Telles , A. M. Valença , A. J. S. Barros , and A. G. da Silva , “Domestic Violence in the COVID‐19 Pandemic: A Forensic Psychiatric Perspective,” Brazilian Journal of Psychiatry 43 (2021): 233–234. 32491034 10.1590/1516-4446-2020-1060 PMC8136389

[11] A. Anurudran , L. Yared , C. Comrie , K. Harrison , and T. Burke , “Domestic Violence Amid COVID‐19,” International Journal of Gynaecology and Obstetrics 150, no. 2 (2020): 255–256. 32472696 10.1002/ijgo.13247 PMC9087628

[12] Z. Momenimovahed , H. Salehiniya , F. Hadavandsiri , L. Allahqoli , V. Günther , and I. Alkatout , “Psychological Distress Among Cancer Patients During COVID‐19 Pandemic in the World: A Systematic Review,” Frontiers in Psychology 12 (2021): 682154. 34650469 10.3389/fpsyg.2021.682154 PMC8506116

[13] D. Dhar , L. McDougal , K. Hay , et al., “Associations Between Intimate Partner Violence and Reproductive and Maternal Health Outcomes in Bihar, India: A Cross‐Sectional Study,” Reproductive Health 15, no. 1 (2018): 109. 29921276 10.1186/s12978-018-0551-2 PMC6009820

[14] E. Berhanie , D. Gebregziabher , H. Berihu , A. Gerezgiher , and G. Kidane , “Intimate Partner Violence During Pregnancy and Adverse Birth Outcomes: A Case‐Control Study,” Reproductive Health 16, no. 1 (2019): 22. 30803448 10.1186/s12978-019-0670-4 PMC6388467

[15] M. Moore , “Reproductive Health and Intimate Partner Violence,” Family Planning Perspectives 31, no. 6 (1999): 302–312. 10614521

[16] P. Tjaden and N. Thoennes , “Prevalence, Incidence, and Consequences of Violence Against Women: Findings From the National Violence Against Women Survey,” NIJ Research in Brief (1998): 172837, https://www.ojp.gov/pdffiles/172837.pdf.

[17] B. Yuksel and F. Ozgor , “Effect of the COVID‐19 Pandemic on Female Sexual Behavior,” International Journal of Gynaecology and Obstetrics 150, no. 1 (2020): 98–102. 32392400 10.1002/ijgo.13193 PMC9087619

[18] N.‐Y. Ko , W.‐H. Lu , Y.‐L. Chen , et al., “Changes in Sex Life Among People in Taiwan During the COVID‐19 Pandemic: The Roles of Risk Perception, General Anxiety, and Demographic Characteristics,” International Journal of Environmental Research and Public Health 17, no. 16 (2020): 5822. 32796759 10.3390/ijerph17165822 PMC7459608

[19] F. Meinck , A. L. Murray , M. P. Dunne , P. Schmidt , G. Nikolaidis , and C. Becan , “Factor Structure and Internal Consistency of the ISPCAN Child Abuse Screening Tool Parent Version (ICAST‐P) in a Cross‐Country Pooled Data Set in Nine Balkan Countries,” Child Abuse & Neglect 115 (2021): 105007. 33721661 10.1016/j.chiabu.2021.105007

[20] A. Fuchs , A. Matonóg , J. Pilarska , et al., “The Impact of COVID−19 on Female Sexual Health,” International Journal of Environmental Research and Public Health 17, no. 19 (2020): 7152. 33007804 10.3390/ijerph17197152 PMC7579227

[21] T. Riley , E. Sully , Z. Ahmed , and A. Biddlecom , “Estimates of the Potential Impact of the COVID‐19 Pandemic on Sexual and Reproductive Health in Low‐ and Middle‐Income Countries,” International Perspectives on Sexual and Reproductive Health 46 (2020): 73–76. 32343244 10.1363/46e9020

[22] R. G. Grose , J. S. Chen , K. A. Roof , S. Rachel , and K. M. Yount , “Sexual and Reproductive Health Outcomes of Violence Against Women and Girls in Lower‐Income Countries: A Review of Reviews,” Journal of Sex Research 58, no. 1 (2021): 1–20. 31902238 10.1080/00224499.2019.1707466

[23] M. Gharacheh , M. E. Kalan , N. Khalili , and F. Ranjbar , “An Increase in Cesarean Section Rate During the First Wave of COVID‑19 Pandemic in Iran,” BMC Public Health 23 (2023): 936. 37226119 10.1186/s12889-023-15907-1 PMC10206354

[24] E. Szilassy , E. C. Barbosa , S. Dixon , et al., “PRimary Care Response to Domestic Violence and Abuse in the COvid‐19 PanDEmic (PRECODE): Protocol of a Rapid Mixed‐Methods Study in the UK,” BMC Family Practice 22, no. 1 (2021): 91. 33980165 10.1186/s12875-021-01447-3 PMC8115859

[25] M. Gharacheh , N. Khalili , M. Ebrahimi Kalan , M. Heidarzadeh , and F. Ranjbar , “Pregnancy‐Related Complications During the COVID‐19 Pandemic in Iran,” Archives of Iranian medicine 27, no. 1 (2024): 30–35. 38431958 10.34172/aim.2024.05 PMC10915933

[26] S. Soltani , A. Mobarakabadi , M. Hosseinpour Kohshahi , et al., “Identifying the Risk Factors of Adverse Pregnancy Outcomes Among Women With COVID‐19: A Population‐Based Case‐Control Study in Southern Iran,” Journal of Caring Sciences 13, no. 2 (2024): 106–115. 39318730 10.34172/jcs.33156 PMC11417295

[27] C. Guo , M. Wan , Y. Wang , et al., “Associations Between Intimate Partner Violence and Adverse Birth Outcomes During Pregnancy: A Systematic Review and Meta‐Analysis,” Frontiers in Medicine 10 (2023): 1140787. 37265489 10.3389/fmed.2023.1140787 PMC10230039

[28] M. A. Straus , S. L. Hamby , S. Boney‐McCoy , and D. B. Sugarman , “The Revised Conflict Tactics Scales (CTS2) Development and Preliminary Psychometric Data,” Journal of Family Issues 17, no. 3 (1996): 283–316.

[29] H. E. Ardabily , Z. B. Moghadam , M. Salsali , F. Ramezanzadeh , and S. Nedjat , “Prevalence and Risk Factors for Domestic Violence Against Infertile Women in an Iranian Setting,” International Journal of Gynaecology and Obstetrics 112, no. 1 (2011): 15–17. 20961542 10.1016/j.ijgo.2010.07.030

[30] M. A. Straus , *Scoring the CTS2 and CTSPC* (Family Research Laboratory, University of New Hampshire, 2004).

[31] M. Gharacheh , T. Sadeghi , M. Mirghafourvand , S. Montazeri , S. Jahanfar , and F. Ranjbar , “Domestic Violence Against Iranian Women During the COVID‐19 Lockdown: A Cross‐Sectional Study,” Health Science Reports 6, no. 10 (2023): e1627. 37829503 10.1002/hsr2.1627 PMC10565101

[32] E. Y. Tenkorang , “Intimate Partner Violence and the Sexual and Reproductive Health Outcomes of Women in Ghana,” Health Education & Behavior 46, no. 6 (2019): 969–980. 31319724 10.1177/1090198119859420

[33] J. G. Silverman and A. Raj , “Intimate Partner Violence and Reproductive Coercion: Global Barriers to Women's Reproductive Control,” PLoS Medicine 11, no. 9 (2014): e1001723. 25226396 10.1371/journal.pmed.1001723 PMC4165751

[34] T. I. Mukherjee , A. G. Khan , A. Dasgupta , and G. Samari , “Reproductive Justice in the Time of COVID‐19: A Systematic Review of the Indirect Impacts of COVID‐19 on Sexual and Reproductive Health,” Reproductive Health 18, no. 1 (2021): 252. 34930318 10.1186/s12978-021-01286-6 PMC8686348

[35] A. K. Normann , A. Bakiewicz , F. Kjerulff Madsen , K. S. Khan , V. Rasch , and D. S. Linde , “Intimate Partner Violence and Breastfeeding: A Systematic Review,” BMJ Open 10, no. 10 (2020): e034153. 10.1136/bmjopen-2019-034153 PMC7783610 33130559

[36] R. S. Mezzavilla , M. F. Ferreira , C. C. Curioni , A. C. Lindsay , and M. H. Hasselmann , “Intimate Partner Violence and Breastfeeding Practices: A Systematic Review of Observational Studies,” Jornal de Pediatria 94 (2018): 226–237. 28888613 10.1016/j.jped.2017.07.007

[37] E. S. Misch and K. M. Yount , “Intimate Partner Violence and Breastfeeding in Africa,” Maternal and Child Health Journal 18, no. 3 (2014): 688–697. 23807715 10.1007/s10995-013-1294-x

[38] A. McFadden , A. Gavine , M. J. Renfrew , et al., “Support for Healthy Breastfeeding Mothers With Healthy Term Babies,” Cochrane Database of Systematic Reviews 2017, no. 2 (2017): CD001141. 10.1002/14651858.CD001141.pub5 PMC6464485 28244064

[39] S. Gandhi , S. Rovi , M. Vega , M. S. Johnson , J. Ferrante , and P.‐H. Chen , “Intimate Partner Violence and Cancer Screening Among Urban Minority Women,” Journal of the American Board of Family Medicine 23, no. 3 (2010): 343–353. 20453180 10.3122/jabfm.2010.03.090124

[40] L. Cadman , J. Waller , L. Ashdown‐Barr , and A. Szarewski , “Barriers to Cervical Screening in Women Who Have Experienced Sexual Abuse: An Exploratory Study,” Journal of Family Planning and Reproductive Health Care 38, no. 4 (2012): 214–220. 23027982 10.1136/jfprhc-2012-100378 PMC3470431

[41] K. L. Levinson , A. M. Jernigan , S. A. Flocke , et al., “Intimate Partner Violence and Barriers to Cervical Cancer Screening: A Gynecologic Oncology Fellow Research Network Study,” Journal of Lower Genital Tract Disease 20, no. 1 (2016): 47–51. 26704329 10.1097/LGT.0000000000000153 PMC4693628

[42] N. Mohammadi , H. E. Kochak , and M. Gharacheh , “The Lived Experience of Domestic Violence in Iranian HIV‐Infected Women,” Global Journal of Health Science 7, no. 5 (2015): 43. 26156897 10.5539/gjhs.v7n5p43 PMC4803845

[43] L. Dhakal , A. R. Aro , and G. Berg‐Beckhoff , “Intimate Partner Violence (Physical and Sexual) and Sexually Transmitted Infection: Results From Nepal Demographic Health Survey 2011,” International Journal of Women's Health 6 (2014): 75. 10.2147/IJWH.S54609 PMC3901740 24470776

[44] S. Bondade , A. Hosthota , K. N. Karthik , and R. Raj , “Intimate Partner Violence, Anxiety, and Depression in Women With Sexually Transmitted Infections—A Hospital‐Based Case Control Study,” Journal of Psychosexual Health 3, no. 1 (2021): 65–72.

[45] M. Gharacheh , N. Mohammadi , F. Ranjbar , H. Emadi Kochak , and S. Montazeri , “A Phenomenological Study of the Experience of Domestic Violence in Iranian Women With HIV,” Journal of Biosocial Science 52, no. 2 (2020): 168–183. 31138339 10.1017/S0021932019000336

[46] M. Meinhart , L. Vahedi , S. E. Carter , C. Poulton , P. Mwanze Palaku , and L. Stark , “Gender‐Based Violence and Infectious Disease in Humanitarian Settings: Lessons Learned From Ebola, Zika, and COVID‐19 to Inform Syndemic Policy Making,” Conflict and Health 15, no. 1 (2021): 84. 34801062 10.1186/s13031-021-00419-9 PMC8605893

[47] A. Peterman , A. Potts , M. O'Donnell , et al., *Pandemics and Violence Against Women and Children* (Center for Global Development, 2020).

